# The one-dimensional organic inorganic hybrid compound poly[(diethyl­ene­triamine)tetra-μ-iodido-dilead(II)]

**DOI:** 10.1107/S1600536808013913

**Published:** 2008-06-13

**Authors:** E. Samet Kallel, H. Boughzala, A. Driss, Y. Abid

**Affiliations:** a Laboratoire de Physique appliquée (LPA), Faculté des Sciences de Sfax, 3018, BP 802, Tunisia; b Laboratoire de Matériaux et Cristallochimie, Institut préparatoire aux études ingénieur de Nabeul, 8000 Mrezga, Nabeul, Tunisia; c Laboratoire de Cristallochimie et des Matériaux, Faculté des Sciences de Tunis, Tunisia

## Abstract

A new organic–inorganic hybrid, [Pb_2_I_4_(C_4_H_13_N_3_)]_*n*_, was obtained by the reaction of C_4_N_3_H_10_ and PbI_2_ at room temperature. The structure is a three-dimensional polymer resulting from the association of PbI_6_ octa­hedra and a mixed lead organic–inorganic PbI_4_(C_4_N_3_H_13_) coordination polyhedron. Both Pb atoms, two I atoms and one N atom lie on a mirror plane. N—H⋯I hydrogen bonds further connect the organic unit and some I atoms.

## Related literature

For related literature, see: Lode & Krautscheild (2001 ▶); Krautscheild *et al.* (2001 ▶); Papavassiliou *et al.* (1999 ▶); Wang *et al.* (1995 ▶); Zhu *et al.* (2004 ▶). 
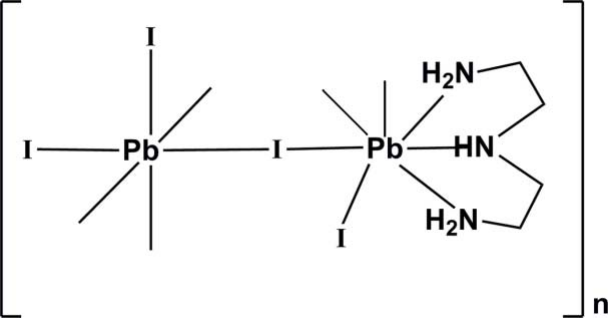


## Experimental

### 

#### Crystal data

[Pb_2_I_4_(C_4_H_13_N_3_)]
*M* *_r_* = 1025.15
Orthorhombic, 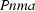

*a* = 17.034 (6) Å
*b* = 9.218 (3) Å
*c* = 11.092 (4) Å
*V* = 1741.6 (10) Å^3^
*Z* = 4
Mo *K*α radiation
μ = 26.38 mm^−1^
*T* = 293 (2) K
0.2 × 0.05 × 0.05 mm

#### Data collection

Enraf–Nonius CAD-4 diffractometer
Absorption correction: ψ scan (North *et al.*, 1968 ▶) *T*_min_ = 0.095, *T*_max_ = 0.268
2906 measured reflections
1998 independent reflections
1202 reflections with *I* > 2σ(*I*)
*R*_int_ = 0.045
2 standard reflections frequency: 120 min intensity decay: 5%

#### Refinement

*R*[*F* ^2^ > 2σ(*F* ^2^)] = 0.040
*wR*(*F* ^2^) = 0.099
*S* = 1.00
1998 reflections
67 parameters
H-atom parameters constrained
Δρ_max_ = 1.57 e Å^−3^
Δρ_min_ = −1.58 e Å^−3^

### 

Data collection: *CAD-4 EXPRESS* (Duisenberg, 1992 ▶; Macíček & Yordanov, 1992 ▶); cell refinement: *CAD-4 EXPRESS*; data reduction: *XCAD4* (Harms & Wocadlo, 1995 ▶); program(s) used to solve structure: *SHELXS97* (Sheldrick, 2008 ▶); program(s) used to refine structure: *SHELXL97* (Sheldrick, 2008 ▶); molecular graphics: *PLATON* (Spek, 2003 ▶) and *DIAMOND* (Brandenburg, 2006 ▶); software used to prepare material for publication: *SHELXL97*.

## Supplementary Material

Crystal structure: contains datablocks I, global. DOI: 10.1107/S1600536808013913/dn2346sup1.cif

Structure factors: contains datablocks I. DOI: 10.1107/S1600536808013913/dn2346Isup2.hkl

Additional supplementary materials:  crystallographic information; 3D view; checkCIF report

## Figures and Tables

**Table 1:** Hydrogen-bond geometry (Å, °)

*D*—H⋯*A*	*D*—H	H⋯*A*	*D*⋯*A*	*D*—H⋯*A*
N1—H1*A*⋯I1^i^	0.90	2.93	3.791 (12)	160
N1—H1*B*⋯I2^ii^	0.90	2.88	3.746 (12)	163
N2—H2⋯I2^iii^	0.91	3.19	3.731 (17)	121

Symmetry codes: (i) 
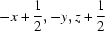
; (ii) 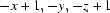; (iii) 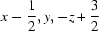.

**Fig. 1.**
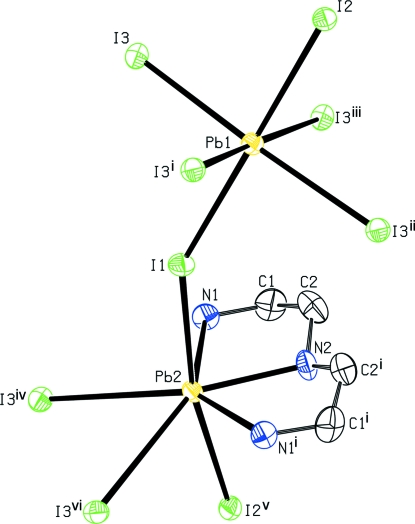
: View of the C4N3H13Pb2I4 with the atom-labelling scheme. Displacement ellipsoids are drawn at the 30% probability level. H atoms have been omitted for clarity. [Symmetry codes: (i)x, 1/2 - y, z; (ii) 1 - x, 1/2 + y,1 - z; (iii) 1 - x, –Y, 1 - z; (iv) 1/2 - x, -y, 1/2 + z; (v) x - 1/2,1/2 - y, 3/2 - z; (vi) 1/2 - x, 1/2 + y, 1/2 + z

**Fig. 2.**
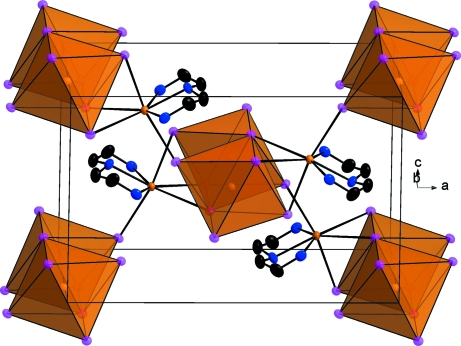
: Packing view of C4N3H13Pb2I4 viewed along [010] direction. H atoms have been omitted for clarity.

## supplementary crystallographic information

### Comment

The basic structure building block of this compound is made up of lead iodide octahedral [PbI_6_] and a mixed lead organic-inorganic PbI_4_(C_4_N_3_H_13_) coordination polyhedron (Fig 1). Both Pb atoms , two I atoms and one N atom lie on a mirror plane. Atom Pb1 is located in the octahedral cavity of the inorganic chains while Pb2 is responsible of the connectivity between organic moiety and inorganic chains. To our knowledge, this is the first report of an organic-inorganic hybrid exhibiting this kind of lead connectivity.

A part of the inorganic back bone is staked as single chains of edge sharing Pb1I_6_ octahedra, as shown in Fig 2. The halide atoms I3 are responsible for the edge sharing between Pb1I_6_ neighboring octahedron to join infinite one dimensional chain (parallel to *b* axis). Within the octahedra the bond lengths around Pb1 range from 3.182 (2) to 3.319 (2) Å which indicate the dominant ionic character of the Pb—I bonds in the inorganic chains. The bond angles I—Pb1—I deviate slightly from ideal octahedral value of 90° and 180°, ranging from 88.39 (4)° to 92.98 (5)° for the adjacent iodides and from 176.76 (3)° to 179.59 (4)° for the opposite ones. This ideal octahedron indicates the unstereochemical activity of lead (II) lone pair electrons (Wang *et al.* 1995). Note that the regular octahedrons are a characteristic feature often encountered in the low dimensional lead iodide structures (Zhu *et al.*, 2004).

As mentioned above, C_4_N_3_H_13_ is in combination with inorganic moiety by three Pb2—N non covalent interaction and hydrogen bonds. The non centrosymmetric organic molecule is slightly twisted around the C—C bond as reflected by a torsion angle of 25.1 (14)°. Bond distances and angles appear to be within normal range.

It is note worthy that the yellow color observed for the title compound is in good accordance with a low dimensional network of lead octahedral (Lode *et al.*, 2001; Krautscheild *et al.*, 2001; Papavassiliou *et al.*, 1999).

### Experimental

An aqueous solution of HI was added to the diethylentriamin to synthesis C_4_H_13_N_3_I_3_ salts. Under ambient conditions, stoechiometric amounts of C_4_H_13_N_3_I_3_ and PbI_2_ with excess HI (to improve PbI_2_ solubility), were sailed in DMF. The resulting solution was kept at room temperature. Yellow needle-like crystals are obtained five weeks later.

### Refinement

All H atoms attached to C atoms and N atom were fixed geometrically and treated as riding with C—H = 0.97 Å (CH_2_) and N—H = 0.90Å (NH_2_) or 0.91Å (NH) 0.86 Å with U_iso_(H) = 1.2U_eq _(C or N).

### Crystal data

**Table:**

[Pb_2_I_4_(C_4_H_13_N_3_)]	*F*_000_ = 1736
*M**_r_* = 1025.15	*D*_x_ = 3.910 Mg m^−^^3^
Orthorhombic, *P**n**m**a*	Mo *K*α radiation λ = 0.71073 Å
Hall symbol: -P 2ac 2n	Cell parameters from 25 reflections
*a* = 17.034 (6) Å	θ = 10.7–13.8º
*b* = 9.218 (3) Å	µ = 26.38 mm^−^^1^
*c* = 11.092 (4) Å	*T* = 293 (2) K
*V* = 1741.6 (10) Å^3^	Needle, yellow
*Z* = 4	0.2 × 0.05 × 0.05 mm

### Data collection

**Table:**

Enraf–Nonius CAD-4 diffractometer	*R*_int_ = 0.045
Radiation source: fine-focus sealed tube	θ_max_ = 27.0º
Monochromator: graphite	θ_min_ = 2.2º
*T* = 293(2) K	*h* = −1→21
Non–profiled ω/2θ scans	*k* = −11→3
Absorption correction: ψ scan(North *et al.*, 1968)	*l* = −1→14
*T*_min_ = 0.095, *T*_max_ = 0.268	2 standard reflections
2906 measured reflections	every 120 min
1998 independent reflections	intensity decay: 5%
1202 reflections with *I* > 2σ(*I*)	

### Refinement

**Table:**

Refinement on *F*^2^	Secondary atom site location: difference Fourier map
Least-squares matrix: full	Hydrogen site location: inferred from neighbouring sites
*R*[*F*^2^ > 2σ(*F*^2^)] = 0.040	H-atom parameters constrained
*wR*(*F*^2^) = 0.099	*w* = 1/[σ^2^(*F*_o_^2^) + (0.0394*P*)^2^] where *P* = (*F*_o_^2^ + 2*F*_c_^2^)/3
*S* = 1.00	(Δ/σ)_max_ < 0.001
1998 reflections	Δρ_max_ = 1.57 e Å^−^^3^
67 parameters	Δρ_min_ = −1.58 e Å^−^^3^
Primary atom site location: structure-invariant direct methods	Extinction correction: none

### Special details

**Table:**

Experimental. Number of psi-scan sets used was 4 Theta correction was applied. Averaged transmission function was used. No Fourier smoothing was applied (North *et al.*,1968).
Geometry. All esds (except the esd in the dihedral angle between two l.s. planes) are estimated using the full covariance matrix. The cell esds are taken into account individually in the estimation of esds in distances, angles and torsion angles; correlations between esds in cell parameters are only used when they are defined by crystal symmetry. An approximate (isotropic) treatment of cell esds is used for estimating esds involving l.s. planes.
Refinement. Refinement of *F*^2^ against ALL reflections. The weighted *R*-factor *wR* and goodness of fit *S* are based on *F*^2^, conventional *R*-factors *R* are based on *F*, with *F* set to zero for negative *F*^2^. The threshold expression of *F*^2^ > σ(*F*^2^) is used only for calculating *R*-factors(gt) *etc*. and is not relevant to the choice of reflections for refinement. *R*-factors based on *F*^2^ are statistically about twice as large as those based on *F*, and *R*- factors based on ALL data will be even larger.

### Fractional atomic coordinates and isotropic or equivalent isotropic displacement parameters (Å^2^)

**Table:**

	*x*	*y*	*z*	*U*_iso_*/*U*_eq_	
Pb1	0.49490 (3)	0.2500	0.49379 (7)	0.0376 (2)	
Pb2	0.23035 (4)	0.2500	0.86880 (7)	0.0361 (2)	
I1	0.32678 (7)	0.2500	0.61874 (12)	0.0482 (4)	
I2	0.66724 (7)	0.2500	0.36339 (11)	0.0441 (3)	
I3	0.43945 (5)	−0.00127 (10)	0.31479 (9)	0.0458 (3)	
N1	0.2835 (7)	−0.0102 (10)	0.8986 (11)	0.049 (3)	
H1A	0.2469	−0.0639	0.9365	0.059*	
H1B	0.2922	−0.0506	0.8260	0.059*	
N2	0.3598 (9)	0.2500	0.9763 (17)	0.055 (5)	
H2	0.3467	0.2500	1.0558	0.066*	
C2	0.4032 (8)	0.113 (2)	0.9595 (18)	0.079 (6)	
H2C	0.4281	0.1143	0.8807	0.094*	
H2D	0.4445	0.1075	1.0195	0.094*	
C1	0.3559 (10)	−0.0133 (16)	0.9684 (18)	0.076 (6)	
H1C	0.3426	−0.0282	1.0525	0.091*	
H1D	0.3867	−0.0961	0.9425	0.091*	

### Atomic displacement parameters (Å^2^)

**Table:**

	*U*^11^	*U*^22^	*U*^33^	*U*^12^	*U*^13^	*U*^23^
Pb1	0.0375 (4)	0.0355 (4)	0.0399 (4)	0.000	0.0008 (3)	0.000
Pb2	0.0285 (3)	0.0402 (4)	0.0397 (4)	0.000	0.0006 (3)	0.000
I1	0.0465 (7)	0.0557 (8)	0.0425 (8)	0.000	0.0098 (7)	0.000
I2	0.0429 (7)	0.0507 (8)	0.0388 (7)	0.000	0.0023 (6)	0.000
I3	0.0484 (5)	0.0472 (6)	0.0419 (5)	−0.0063 (4)	−0.0028 (4)	−0.0008 (5)
N1	0.060 (7)	0.025 (6)	0.062 (9)	0.006 (5)	0.010 (6)	0.001 (6)
N2	0.043 (8)	0.062 (12)	0.059 (12)	0.000	−0.015 (9)	0.000
C2	0.048 (9)	0.084 (14)	0.103 (16)	0.013 (10)	−0.007 (10)	0.011 (13)
C1	0.088 (12)	0.043 (10)	0.096 (16)	0.017 (9)	−0.023 (12)	0.001 (11)

### Geometric parameters (Å, °)

**Table:**

Pb1—I1	3.1816 (17)	N1—H1A	0.9000
Pb1—I3	3.1936 (13)	N1—H1B	0.9000
Pb1—I2	3.2725 (16)	N2—C2	1.476 (18)
Pb1—I3^i^	3.3190 (13)	N2—H2	0.9100
Pb2—N2	2.506 (15)	C2—C1	1.42 (2)
Pb2—N1	2.585 (10)	C2—H2C	0.9700
Pb2—I2^ii^	3.1591 (18)	C2—H2D	0.9700
Pb2—I1	3.2236 (17)	C1—H1C	0.9700
Pb2—I3^iii^	3.7392 (17)	C1—H1D	0.9700
N1—C1	1.457 (19)		
			
I1—Pb1—I3	90.26 (3)	Pb1^ix^—I3—Pb2^viii^	74.61 (2)
I3—Pb1—I3^iv^	92.98 (5)	Pb1—I1—Pb2	146.46 (5)
I1—Pb1—I2	179.59 (4)	Pb2^x^—I2—Pb1	83.66 (4)
I3—Pb1—I2	89.46 (3)	Pb1—I3—Pb1^ix^	90.21 (4)
I1—Pb1—I3^i^	91.41 (3)	C1—N1—Pb2	112.5 (8)
I3—Pb1—I3^i^	176.76 (3)	C1—N1—H1A	109.1
I3^iv^—Pb1—I3^i^	89.79 (4)	Pb2—N1—H1A	109.1
I2—Pb1—I3^i^	88.88 (3)	C1—N1—H1B	109.1
I3—Pb1—I3^v^	89.79 (4)	Pb2—N1—H1B	109.1
I3^iv^—Pb1—I3^v^	176.76 (3)	H1A—N1—H1B	107.8
I3^i^—Pb1—I3^v^	87.39 (4)	C2—N2—C2^iv^	117.7 (17)
N2—Pb2—N1	68.4 (3)	C2—N2—Pb2	112.4 (9)
N1^iv^—Pb2—N1	136.3 (5)	C2—N2—H2	104.2
N2—Pb2—I2^ii^	81.5 (4)	Pb2—N2—H2	104.2
N1—Pb2—I2^ii^	89.9 (3)	C1—C2—N2	114.1 (12)
N2—Pb2—I1	87.8 (4)	C1—C2—H2C	108.7
N1—Pb2—I1	86.1 (3)	N2—C2—H2C	108.7
I2^ii^—Pb2—I1	169.26 (4)	C1—C2—H2D	108.7
I3^iii^—Pb2—N1	73.9 (3)	N2—C2—H2D	108.7
I1—Pb2—I3^iii^	104.85 (3)	H2C—C2—H2D	107.6
I3^iii^—Pb2—N2	139.17 (15)	C2—C1—N1	115.4 (13)
I3^iii^—Pb2—I3^vi^	75.64 (2)	C2—C1—H1C	108.4
I2^vii^—Pb2—I3^iii^	83.54 (3)	N1—C1—H1C	108.4
I3^vi^—Pb2—N1	149.3 (3)	C2—C1—H1D	108.4
I3^vi^—Pb2—N1^iv^	73.8 (3)	N1—C1—H1D	108.4
Pb1—I3—Pb2^viii^	125.02 (3)	H1C—C1—H1D	107.5
			
C1—N1—N2—C2	25.1 (14)		

Symmetry codes: (i) −*x*+1, *y*+1/2, −*z*+1; (ii) *x*−1/2, *y*, −*z*+3/2; (iii) −*x*+1/2, −*y*, *z*+1/2; (iv) *x*, −*y*+1/2, *z*; (v) −*x*+1, −*y*, −*z*+1; (vi) −*x*+1/2, *y*+1/2, *z*+1/2; (vii) *x*−1/2, −*y*+1/2, −*z*+3/2; (viii) −*x*+1/2, −*y*, *z*−1/2; (ix) −*x*+1, *y*−1/2, −*z*+1; (x) *x*+1/2, *y*, −*z*+3/2.

### Hydrogen-bond geometry (Å, °)

**Table:**

*D*—H···*A*	*D*—H	H···*A*	*D*···*A*	*D*—H···*A*
N1—H1A···I1^xi^	0.90	2.93	3.791 (12)	160
N1—H1B···I2^v^	0.90	2.88	3.746 (12)	163
N2—H2···I2^ii^	0.91	3.19	3.731 (17)	121

Symmetry codes: (xi) −*x*+1/2, −*y*, *z*+1/2; (v) −*x*+1, −*y*, −*z*+1; (ii) *x*−1/2, *y*, −*z*+3/2.

## References

[bb1] Brandenburg, K. (2006). *DIAMOND* Crystal Impact GbR, Bonn, Germany.

[bb2] Duisenberg, A. J. M. (1992). *J. Appl. Cryst.* **25**, 92–96.

[bb3] Harms, K. & Wocadlo, S. (1995). *XCAD4* University of Marburg, Germany.

[bb4] Krautscheild, K., Lode, C., Vielsack, F. & Vollmer, H. (2001). *J. Chem. Soc. Dalton Trans.* pp. 1099–1104..

[bb5] Lode, C. & Krautscheild, H. (2001). *Z. Anorg. Allg. Chem* **627** 1454–1458.

[bb6] Macíček, J. & Yordanov, A. (1992). *J. Appl. Cryst.* **25**, 73–80.

[bb7] North, A. C. T., Phillips, D. C. & Mathews, F. S. (1968). *Acta Cryst.* A**24**, 351–359.

[bb8] Papavassiliou, G. C., Mousdis, G. A., Raptopoulou, C. P. & Terzis, A. Z. (1999). *Z. Naturforsch. Teil B*, **54**, 1405–1409.

[bb9] Sheldrick, G. M. (2008). *Acta Cryst.* A**64**, 112–122. 10.1107/S0108767307043930 18156677

[bb10] Spek, A. L. (2003). *J. Appl. Cryst.* **36**, 7–13.

[bb11] Wang, S., Mitzi, D. B., Feild, C. A. & Guloy, A. (1995). *J. Am. Chem. Soc.* **117**, 5297–5302.

[bb12] Zhu, X. H., Mercier, N., Allain, M., Frère, P., Blanchard, P., Roncali, J. & Riou, A. (2004). *J. Solid State Chem.* **177**, 1067–1071.

